# Thromboembolic Adverse Drug Reactions in Janus Kinase (JAK) Inhibitors: Does the Inhibitor Specificity Play a Role?

**DOI:** 10.3390/ijms22052449

**Published:** 2021-02-28

**Authors:** Przemysław J. Kotyla, Małgorzata Engelmann, Joanna Giemza-Stokłosa, Bartosz Wnuk, Md Asiful Islam

**Affiliations:** 1 Department of Internal Medicine, Rheumatology and Clinical Immunology, Faculty in Katowice, Medical University of Silesia, 40-635 Katowice, Poland; 2 Department of Physiotherapy in Internal Medicine, Academy of Physical Education in Katowice, 40-065 Katowice, Poland; m.engelmann@awf.katowice.pl; 3 Department of Neurology, Regional Hospital in Oświęcim, 32-600 Oświęcim, Poland; giemza@mp.pl; 4 Department of Rehabilitation, Faculty of Health Sciences in Katowice, Medical University of Silesia, 40-635 Katowice, Poland; bwnuk@sum.edu.pl; 5 Department of Haematology, School of Medical Sciences, Universiti Sains Malaysia, Kubang Kerian 16150, Kelantan, Malaysia

**Keywords:** venous thromboembolism, JAK kinase inhibitor, prothrombotic effect, deep vein thrombosis, JAK/STAT pathway

## Abstract

Recent advances in immunology enabled the characterization of several signal transmitting pathways responsible for proper cytokine and chemokine signaling. Among them, Janus kinases (JAKs) are essential components of receptor activation systems. The discovery of JAK kinases enabled the synthesis of JAK kinase inhibitors (JAKi or Jakinibs), which have proven to be efficacious in the treatment of hematologic malignancies and several rheumatological disorders and continue to be investigated in many clinical indications. Blocking multiple cytokines belonging to several cytokine families with a single small molecule may, however, create a potential risk for the patients. Recently, a higher risk of thromboembolic complications, namely, deep vein thrombosis and pulmonary embolism, has been recognized as the main concern during treatment with Jakinibs. At present, it is not entirely clear whether this increased risk is related to direct cytokine blockade, the presence of concomitant diseases in treated patients or other unknown circumstances that work together to increase the risk of this side effect. In this review, we discuss data on the risk of thromboembolic side effects, with special emphasis on the mechanism that may be responsible for this increased risk. Many indirect data indicate that higher thromboembolic risk may be related to the specificity of JAK inhibitor action, such that preferentially blocking one signaling pathway upsets the balance between pro and anti-thrombotic activities.

## 1. Introduction

Autoimmune diseases have various pathophysiological backgrounds characterized by the aberrant or defective immune response. Despite enormous progress in the last three decades, the precise mechanism that leads to the development of these diseases is still a subject of scientific controversy. Although many mechanisms have been proposed, none is universal and satisfactorily explains the pathological mechanism. Consequently, therapeutic approaches are mainly based on immune suppression that blocks most immune pathways. The introduction of cytokine-targeted therapies for rheumatoid arthritis (RA), psoriasis and inflammatory bowel diseases demonstrated that it is possible to halt disease progression by blocking a single cytokine and subsequently inhibiting the entire cytokine-regulated inflammatory pathway. The use of cytokine blocking therapy with high molecular weight compounds, commonly referred to as biologic or biological disease-modifying anti-rheumatic drugs (bDMARDs), creates many therapy-associated complications. Biologics, as large proteins, often produce allergic reactions and are challenging to produce and handle [1]. Moreover, this may negatively impact patient immunity, predisposing to viral and bacterial infections and causing heart failure and peripheral nervous system damages in predisposed patients [2,3]. Moreover, blockade of one signaling pathway does not necessarily translate to reduction of cytokine concentration in the body [4]. Discovery of the immune pathway that orchestrates immune mechanisms translated directly to the development of new therapeutics. Among several mechanisms that transmit cytokine signals to the nucleus, Janus kinase/signal transducers and activators of transcription (JAK/STAT) pathway is of particular interest. It is responsible for transmitting signals from many cytokines within the same signaling pathway. This pathway was the theoretical background to the discovery of drugs that offer blockade of several cytokines with one small synthetic compound [5,6,7,8]. Following the introduction of tofacitinib (the first FDA-approved JAK inhibitor), several synthetic compounds with potential to block this pathway have been developed and intensively tested in various indications [9]. This group of fully synthetic immune modulators is called JAK inhibitors (JAKi) or Jakinibs and offers the blockade of many cytokines with a single small molecule. Treatment with Jakinibs has proven efficacious, relatively safe and equal or even superior to treatment with conventional biologics [10,11].

As a potent tool that can inhibit many cytokines belonging to several cytokine families thus blocking many pathways, this approach may also be risky. The therapeutic approach based on the blockade of multiple cytokines and interferons may have many pathophysiological consequences as the role of the JAK/STAT pathway in the development of immune diseases has not been completely explained. Moreover, this pathway is a key element of many mostly undercharacterized regulatory networks; therefore, real-world clinical data accumulation is essential.

Recently, a higher risk of thromboembolic complications, namely deep vein thrombosis (DVT) and pulmonary embolism (PE), was reported to be the primary concern during treatment with Jakinibs [12,13,14]. At the moment, it is not entirely clear whether this increased risk is related to direct cytokine blockade, the presence of concomitant diseases in treated patients or other unknown circumstances that work together to increase the risk of this side effect.

In this review, we synthesized data on the risk of thromboembolic side effects; with particular emphasis on the mechanisms that may be responsible for this increased risk.

## 2. JAK/STAT Pathway

**Figure 1:**
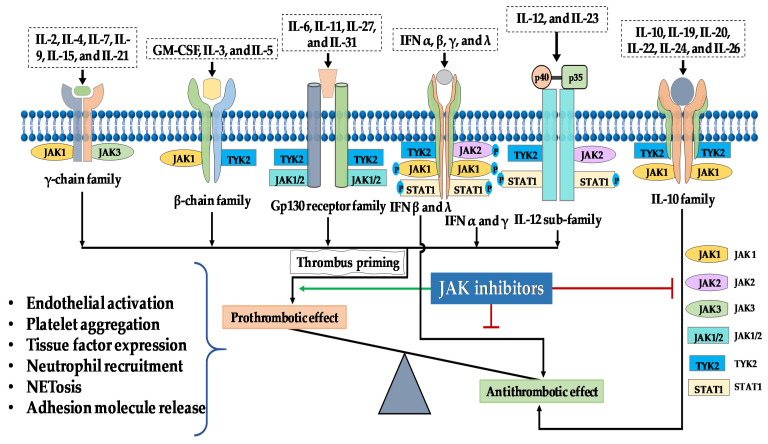
Possible mechanism for the prothrombotic effects associated with the administration of Jakinibs. JAKs in various combinations bind to cytokine receptors that transmit prothrombotic and proinflammatory signals from a wide range of cytokines. With the exception of IL-10, IFNβ and IFNλ that exert anti-thrombotic potential, signaling downstream of these cytokines creates a permissive background for thrombus formation. Non-specific Jakinibs that target both of the IL-10R-associated JAKs (JAK1 and TYK2) or IFNβ and IFNλ associated JAKs (JAK1 and TYK2) may result in an imbalance in the pro- and anti-thrombotic signaling resulting in thrombus priming.

Janus kinases (JAKs) are a sub-family of the tyrosine kinase family consisting of four members, namely JAK 1, JAK2, JAK3 and tyrosine kinase 2 (TYK2). They are non-receptor tyrosine kinase and possess seven distinct homologous regions which fold into four structural domains; the FERM-domain (where F stands for F4.1 protein; E, ezrin; R, radixin, and M, moesin), Src homology 2 (SH2) domain, the pseudo-kinase domain and the kinase domain [15]. Their primary function is to phosphorylate tyrosine residues to activate downstream signaling proteins and transmit signals to the nucleus, thus evoking physiological function. Activation of the system enables transfer of signals from growth factors, cytokines, chemokines and interferons that orchestrate critical processes, including immune response, growth, development and apoptosis [16]. Moreover, as many cytokines, chemokines and growth factors transmit their signals through the JAK/STAT pathway, precise regulation of many normal and patho-physiological processes occurs. Physiological signals from more than 50 ligands are transmitted via the JAK/STAT system, making this pathway a critical element of the immune systems’ central communication system [17]. The four known JAKs working in pairs as homo or heterodimers create multiple JAK combinations, making it difficult to predict the pathophysiological effect when a specific JAK is inhibited (Figure 1). It has been shown that the JAK/STAT system transmits not only signals responsible for driving the inflammatory response, but also acts as a powerful transmitter of anti-inflammatory signals. The final outcome is dependent on which cytokines are predominantly blocked.

It has been established that the majority of known cytokine receptors transmit signals from cytokines, hormones and growth factors to the nucleus using JAKs as a catalytic center to activate transcription of ligand-regulated genes. Various receptor-ligand complexes can activate one specific JAK; thus, one JAK molecule can transmit signals from several cytokines. Many cells express JAK1, JAK2 and TYK2, whilst hematopoietic, myeloid and lymphoid cells predominantly express JAK3 [18]. Following cytokine recognition by a receptor, the receptor-associated JAK becomes activated [19]. Active JAK then phosphorylates tyrosine residues in the cytoplasmic part of the receptor, creating docking sites for STAT and other signaling proteins. Depending on the type of receptor and the position of the phosphotyrosine in the receptor tail, STAT1, STAT2, STAT3, STAT4, STAT5a, STAT5b or STAT6, may be recruited [20]. Active STATs translocate to the nucleus, where they interact with DNA regulatory elements, resulting in changes in the expression of target genes [21,22].

Cytokine receptors are typically divided into two subgroups: class I and class II based on receptor and cytokine homology [23,24,25,26]. The class I receptor family comprises five subfamilies—the homodimeric hormone receptor family, and four families whose receptors pair with gamma chains (γc), beta chains (βc), gp130 or the p40 subunit, respectively. Interleukins (ILs) IL-2, IL-4, IL-7, IL-9, IL-15 and IL-21 signal via γc receptor [27], whilst β chain receptors interact with granulocyte-monocyte colony stimulating factor (GM-CSF), IL-3 and IL-5. The IL-6 cytokine subfamily (IL-6, IL-11, IL-27, IL-31, IL-35, IL-39, ciliary neurotrophic growth factor, oncostatin M, cardiotrophin1 and cardiotrophin-like cytokine factor 1) utilizes gp130 as a receptor component. [28,29,30]. IL-12 and IL-23 also bind to class I receptors containing the common subunit p40 [31,32]. Several hormone-like cytokines such as growth hormone, leptin, erythropoietin and thrombopoietin, also signal via class I receptors.

Many signaling molecules belong to the class II cytokine family. Among them, type I, II and III interferons (IFNs) (IFN-α, IFN-β, IFN-γ, IL-28 and IL-29) and cytokines belonging to the IL-10-related family (IL-10, IL-19, IL-20, IL-22, IL-24 and IL-26) are recognized as the most important ones [25].

JAK1 pairs with the other three JAKs to transmit signals from IL-6 (paired with JAK2 or TYK2), IL-10, IL-19, IL-20, IL-22, (paired with TYK2), IFN-γ (paired with JAK2) IFN-α, IFN-β, and IFN-λ (paired with TYK2) [26]. JAK2 binds to receptors that transduce signals from hormone-like cytokines such as erythropoietin, thrombopoietin, growth hormone, GM-CSF, IL-3 and IL-5 [33]. JAK2 is also a component of JAK heterodimers responsible for signal transmission from IFN-γ (paired with JAK1) and paired with TYK2 to transmit signals from IL-12 and IL23 [34]. JAK3 is attached to the γ chain that transmits signals from IL-2, IL-4, IL-7, IL-9, IL-15 and IL-21 [33]. TYK2 signals downstream of IL-12 and IL-23 (together with JAK2) and type I IFNs (paired with JAK1) [34,35]. Thus, in contrast to biologic-targeted therapy, several cytokines that utilize the same receptor type, and thus JAK-pairing may be blocked by a single Jakinib.

## 3. Thromboembolic Mechanisms

Deep vein thrombosis (DVT) resulting in blood clot formation in deep veins is a severely debilitating disease affecting several million people worldwide [36]. In addition to the local symptoms, clots can migrate to the lungs and obstruct the pulmonary artery leading to a clinical entity known as pulmonary embolism (PE) [37]. DVT and PE together are often referred to as venous thromboembolism (VTE) [38]. Factors predisposing to DVT include conditions such as hereditary factors, hypercoagulability states, connective tissue diseases (mainly antiphospholipid syndrome), cancer, pregnancy, or trauma [39,40,41]. However, in many cases, the development of idiopathic DVT is caused by prolonged immobility leading directly to reduced blood flow predisposing to thrombus formation [42]. Some medications may change the subtle balance between coagulation and fibrinolysis, thus predisposing to thrombosis, for example, estrogens are directly linked to DVT and strokes [43,44]. From a mechanistic point of view, blood clot formation results from coagulation cascade activation that finally leads to fibrin generation.

In situ inflammation, endothelial dysfunction and damage, followed by leukocyte and platelet recruitment are essential steps in the initiation and progression of thrombosis [45,46]. In line with endothelial activation, levels of adhesion molecules rise to enable blood clotting and effector immune cell interaction with the endothelial surface [47,48].

Clinical assessment of patients with DVT is directed towards the identification of risk factors for the disease. Among many factors identified so far, age (linked with increased expression of coagulation factors), major surgery (primarily orthopedic surgery), prolonged immobilization, infections, neoplasms and connective tissue diseases play a role. As typical risk factors cannot explain a substantial proportion of VTE episodes, research has also been directed towards identifying coagulation system abnormalities. Several biochemical and genetic abnormalities have been identified in patients with DVT to date. Among these, positivity for antiphospholipid antibodies, including anticardiolipin antibodies and lupus anticoagulant [49,50], as well as the prothrombin gene mutation G20210A [51], the C677 > T substitution in the methylenetetrahydrofolate reductase gene (a mutation that causes increased plasma levels of homocysteine) [52], and JAK2 and factor V Leiden mutations [53], have been reported to be correlated with DVT risk. Additionally, protein C, protein S and antithrombin III deficiency, activated protein C resistance (APCR) and high levels of factor VIII [54,55] are associated with the development of DVT [56,57].

A notable example of dysregulated gene functioning is a mutation in the JAK2 gene resulting in a gain of function of the molecule that is strongly linked to thromboembolic complications in mutation carriers. JAK2 mutation-related thromboembolic events are part of a more comprehensive concept of so-called clonal hematopoiesis of indeterminate potential (CHIP), in which somatic mutations cause clonal expansion of mutant hematopoietic cells without evident disease development [58]. JAK2 plays a role in controlling hematopoietic precursor cell maintenance and function [59]. Inherited mutations of *JAK2* (the most common is JAK2^V617F^—Janus kinase 2 with valine to phenylalanine substitution on codon 617) are detected in patients with hereditary thrombocytosis [60], while somatic mutations of the gene link to various phenotypes, including erythrocytosis. Moreover, clonal hematopoiesis is observed predominantly in aging humans. These clones are relatively rare in people < 40 years, but their frequency rises to >10% in those 70 years old [61,62]. Parallel to this, aging in humans is linked to a state of chronic, low-grade inflammation mediated by higher concentrations of circulating IL-6 and C-reactive protein [63]. In addition, a substantial subset of elderly individuals showed inflammasome activation and increased IL-1β levels [64]. The direct influence of these pro-inflammatory cytokines on thrombus formation in humans is still under debate, however, data obtained so far showed that both cytokines created a permissive background for the development of DVT [65,66,67].

The role of cytokines, however, should be discussed in the broader context. In a simplified way, cytokines and chemokines may be categorized as either “pro-inflammatory” or “anti-inflammatory”. Pro-inflammatory molecules usually act as strong catalyzers for thrombus formation whilst their anti-inflammatory counterparts exert anti-thrombotic potential [68]. The impact of the cytokine on thrombus formation is somewhat indirect. From the pathophysiological point of view, thrombosis occurs when there is an imbalance in endogenous anticoagulation and hemostasis factors. Among three common factors predisposing to thrombosis: (i) damage to the endothelial lining of the vessel wall; (ii) a hypercoagulable state and (iii) arterial or venous blood stasis, the role of the endothelial damage should be considered in terms of the immune response at the level of the endothelium [69]. To address these changes in the endothelial surface, the term immunothrombosis has been coined recently [70]. In this process, hundreds of factors including cells, cytokines, chemokines and adhesion molecules create the specific milieu resulting in thrombus formation as a part of host defense [71]. All known cytokines may potentially be involved in this process. Some transmit their signals via the JAK/STAT pathway, in particular, IL-6, IL-9, IFNs and anti-inflammatory IL-10 [72]. As these cytokines utilize multiple types of receptors coupled with a variety of JAK/STAT combinations, the downstream effect depends on which cytokines are predominantly expressed.

## 4. The Role of the JAK/STAT Pathway in Thrombus Formation

The JAK/STAT pathway has a role in several diseases, including inflammation, cancer, immunity and immune deficiency [73], and Jakinibs have been shown to modulate inflammation and the immune response [74]. The role of cytokines that transmit their signals via the JAK/STAT pathway in the functioning of the coagulation system is not restricted to a given cytokine’s direct effect, since cytokines act on almost all immunocompetent cells [75]. For example, IL-2, IL-7 and IL-15 are essential factors for the growth and development of T-cells [76] whilst IL-15 and IL-21 regulate B cell and natural killer (NK) cell fate [77,78].

### JAK/STAT and Platelet Function

Several protein tyrosine kinases have been identified to play significant roles in platelet function [79,80], among them JAK3 whose activity is of particular importance as it is constitutively active in human platelets [81,82,83].

Moreover, tight control over platelet function is mediated by IL-9 and IL-21. Feng et al. [84] showed that IL-9 acting via the JAK2/STAT3 pathway is a crucial regulator of platelet function and contributed significantly to DVT development. This was dependent on JAK/STAT activation resulting in enhanced expression of the adhesion molecule P-selectin within platelets and resulted in augmented thrombus formation [84]. IL-21 regulates the function of immune cells such as B, NK and T lymphocytes, macrophages and dendritic cells, and is also involved in vascular endothelial cell homeostasis [85]. With a high proinflammatory potential, IL-21 is linked to the development of autoimmune diseases and inflammatory disorders, and plays a role in reactive thrombocytosis [86] Furthermore, IL-21 was produced by almost all Th1 and Th17/Th1 cells specific for β2GPI, a key target for antiphospholipid antibodies in antiphospholipid syndrome. IL-21 is up-regulated in patients with peripheral artery diseases [87]. Another potential mechanism by which the JAK/STAT pathway may contribute to development of the thromboembolic state is the regulation of thrombopoietin generation and promotion of platelet formation from megakaryocytes [83].

In addition to its canonical transcriptional activity, STAT has activities that are independent of its transcriptional role. Zhou et al. [88], showed that STAT3 in platelets during inflammation might also exert nontranscriptional activity; STAT3 dimers may act as a scaffold and enhance collagen-induced intracellular signaling, resulting in platelet activation, calcium mobilization and activation of IL-6/IL-6R, and probably also IL-15/IL-15R. Following STAT3 activation, STAT molecules interact directly with spleen tyrosine kinase (Syk) and phospholipase C*γ*2 (PLC*γ*2) to form a complex, which enhances the catalytic interaction between Syk kinase and its substrate PLC*γ*2 [88]. Activated PLC*γ*2 hydrolyzes phosphatidylinositol 4,5-bisphosphate to produce inositol 1,4,5-triphosphate, leading to calcium mobilization. This mechanism directly links the nontranscriptional activity of STAT3 with collagen-induced signaling in platelets making platelets hyperactive under inflammatory conditions [83,89]. These data also suggest that due to the proximity of gp130 (a subunit of the IL-6 receptor) and collagen-glycoprotein VI (GPVI), cross-talk between gp130 and GPVI occurs that potentially provides a novel mechanism for inflammation-induced platelet hyperactivity, making the IL-6-gp130-JAK2-STAT3 pathway a potential target to block this hyperactivity [90]. In line with this finding, inhibition of JAK2 could potentially reduce the risk of thrombus formation due to reduced platelet activity.

## 5. The Role of Cytokines in Thrombus Formation

Inflammatory processes are precisely orchestrated by cytokines and chemokines [91]. They have crucial functions in development, differentiation and coordination of immune cells, playing a role in health and disease. It has been suggested that these inflammatory mediators are also essential players in VTE [92].

All known and perhaps not already known cytokines and chemokines create a unique network of self-interactions, activation, and regulatory loops. Any disturbance could result directly in the development of autoimmunity, malignancy, allergy, immunodeficiency, or immunothrombosis. Therefore, the clinical effect of Jakinbs depends on the extent to which pro and anti- inflammatory pathways are blocked by any particular Jakinib.

**Figure 2:**
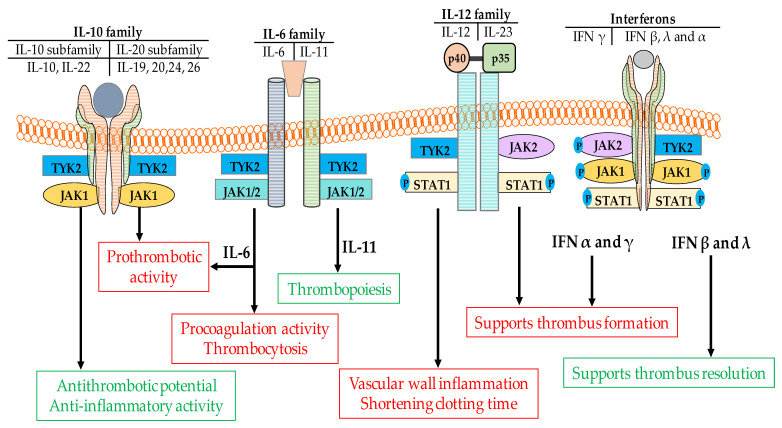
Diverse role of cytokines in thrombus formation. Cytokines belonging to the IL-10 family exert different functions; the three anti-inflammatory cytokines (IL-10, IL-19, IL-22) exert anti-thrombotic potential whilst other cytokines in family (namely IL-20, IL-24, IL-26) show strong proinflammatory activity. These pro and anti-inflammatory activities are transmitted via different JAK pairings; JAK1/TYK2 and JAK1/JAK2 for pro and anti-thrombotic activity respectively. Targeted inhibition of a given JAK can thus either block or augment the prothrombotic activity. Proinflammatory cytokines from the IL6 and IL-12 families also signal through JAK1, JAK2 and TYK2, thus administration of Jakinibs may block pro-inflammatory (pro-thrombotic) signals downstream of these cytokines. The more diverse type of signaling is observed in IFN family. IFN-α and IFN-γ exert strong proinflammatory potential, while IFN-β and IFN-λ show anti-inflammatory activity. Among all four types of INFs only IFN-γ is signaling via JAK1/JAK2 pair The remaining ones utilize JAK1/TYK2 pair. This shows that type of signaling (pro or anti-inflammatory) is unrelated to JAK composition, but the clinical effect depends on the extent to which the pro- and anti-inflammatory pathways are blocked by any particular Jakinib.

There is a close relationship between a given cytokine’s inflammatory activity and its role in thrombus formation [93]. Therefore, inhibiting the JAK/STAT pathway that contributes to a particular cytokine’s activity may translate to a reduction of cytokine-driven thromboembolic risk (Figure 1). Unfortunately, real-world data indicated that this is not entirely the case. Depending on the physiological function, the role of particular cytokines may be recognized in the light of either pro or anti-thrombotic activities. With the exception of IL-10, IL-19, IFN-β and IFN-λ and to a lesser degree IL-22, the other cytokines such as IFN-α, IFN-γ, IL-6, IL-9, IL-12 and IL-23 are strong proinflammatory cytokines and exert strong prothrombotic potential (Figure 2 and Table 1).

### 5.1. Interferons

IFN-γ and IFN-α are recognized as the strong proinflammatory players, and the role of IFNs in the development of several autoimmune disorders has been confirmed [94,95,96,97]. In contrast, IFN-λ and IFN-β exert anti-inflammatory potential and they are recognized as anti-thrombotic cytokines [98,99]. Inhibition of IFN-γ signaling via JAK/STAT blockade may reduce the severity of several diseases [100,101,102]. IFNs are deeply involved in thrombus formation [72]. IFN-γ may increase prothrombotic activity via targeting matrix metalloproteinase-9 (MMP-9), and vascular endothelial growth factor (VEGF) gene expression, however, it does not influence natural anti-thrombotic agents such as tissue plasminogen activator, urokinase and plasminogen activator inhibitor type-1. Moreover, it exerts a potent influence on collagen formation. At the moment, there is no direct data on how Jakinibs may influence IFN-dependent thrombus formation. We may only suppose that the result might be similar to direct IFN inhibition when an IFN specific antibody is administered. In a mouse model, IFN-γ blockade resulted in high expression of MMP-9 and VEGF that translated directly to accelerated thrombus resolution [103].

It is therefore hypothesized that JAK inhibition downstream of IFN-α and IFN-γ may augment thrombus resolution. As shown by Luther et al., substantial amounts of IFNs are produced locally by the memory T-cells that infiltrate the vascular wall [104]. Inhibition of the JAK/STAT pathway may reduce signaling from IL-7 that is required for normal growth and development of T cells [105], thus reducing vascular inflammation and reducing thrombus formation. François-René Bertin and his team [106] demonstrated that NK cell-derived IFN-γ is an essential factor in promoting neutrophil extracellular trap (NET) formation in mice during DVT. In the microcirculation, NETs are one of the components initiating the formation of microthrombi [107]. Less is known on IFN-β and IFN-λ in this regard. Clinical and experimental data suggest anti-thrombotic activity of these cytokine [98,99]. Taken together, these data suggest that reducing the IFNs activity may, therefore, exert diverse effects on the progression of thrombus formation [103].

### 5.2. Interleukin-6

The role of cytokines in the development of VTE is not limited to IFNs. The pro-inflammatory cytokine-IL-6, is also involved in prothrombotic activity [108,109,110]. The role of IL-6 activity in thrombus formation in humans has been recently confirmed, and IL-6 levels are elevated in VTE and prothrombotic syndromes [65,66]. Importantly targeting IL-6 may reduce vein wall hypertrophy and collagen deposition [111]. IL-6 stimulates tissue factor (TF) synthesis and thrombopoietin expression in the liver, thus acting simultaneously on two arms of thrombus formation [112]. However, it is unclear whether targeting the JAK/STAT system will have the same effect as direct reduction of IL-6 activity. 

### 5.3. Interleukin-9

The IL-9 receptor is a heterodimer composed of the IL-9 receptor α-chain (IL-9Rα) and the common γ chain [113]. The role of IL-9 in immune system functioning is unusual. Depending on the type of cells it acts on, IL-9 can positively or negatively impact immune responses. IL-9 is produced by mast cells, NK cells, T-cells, Th9, Th17 and regulatory T (Treg) cells [114]. IL-9’s pro-inflammatory role consists of the facilitation of mast cell expansion and enhancement of the action of several pro-inflammatory cytokines such as IL-1β, IL-5, IL-6, IL-9, and IL-13. Acting on Th17 cells, it promotes Th17 cell proliferation and may also enhance Treg’s suppressive activity. IL-9 targets antigen presenting cells (APCs) to promote TGF-β production and downregulate IL-12 expression. As a potent regulator of the immune response, the role of IL-9 in VTE is likely to be indirect. A recent study [115] suggests that it may be realized via regulation of IL-17 activity, promoting arterial and venous thrombus formation by acting on platelet activation. Feng et al. [84] measured higher IL-9 and p-selectin plasma levels in VTE patients. Moreover, in this study, IL-9 had a stimulatory effect on platelet aggregation, p-selectin expression and JAK2/STAT3 pathway activation.

### 5.4. Interleukin-12/23

IL-12 and IL-23 are pro-inflammatory cytokines that are involved in several autoimmune disorders, including psoriasis, psoriatic arthritis and inflammatory bowel disease [116]. These cytokines belong to the IL-12 cytokine family. Both cytokines are heterodimers and are formed of two subunits. IL-12 is composed of the IL-12p35 and the IL-12/IL-23p40 subunits whilst IL-23 consists of the IL-23p19 subunit bound to the IL-12/IL-23p40 subunit. Both cytokines bind to IL-12Rβ receptor via their common p40 subunit. Despite their similar structures, these cytokines transmit different signals. IL-12 acts via the JAK2, Tyk2/STAT 4 pathway to transmit signals promoting the synthesis of IFN-γ and Th1 cell polarization. IL-23 activates STAT3 and STAT4 to polarize Th cells toward a Th17 response with subsequent synthesis of Th-17 dependent cytokines (IL-17A, IL-17F and IL-22) [116]. The role of IL-12 dependent activation on thrombus formation has been confirmed in a stenosis model of DVT. IL-12 released by myelomonocytic cells supports IFN-γ production by T_EM_ cells responsible for the infiltration of inflammatory monocytes into thrombotic veins and subsequent thrombus formation [104]. Recently a crucial role for IL-12 in thrombus formation was confirmed in a mouse model where inhibition of an immune cell transcription factor, T-bet, in myelomonocytic cells suppressed IL-12 synthesis and fostered thrombus resolution [117]. Similar conclusions came from animal studies on IL-23. Inhibition of IL-23 activity lengthened time to occlusive venous formation in a mouse model of psoriasis suggesting IL-23 involvement in thrombus formation [118]. In human studies, high IL-23 levels and increased activity of the IL23/IL-17 axis were found in primary antiphospholipid syndrome suggesting a role for this cytokine in autoimmune thrombosis development [119,120].

### 5.5. Interleukin-10

The prototypical anti-inflammatory cytokine IL-10, targets both the innate and adaptive immune systems. It has been hypothesized that IL-10’s anti-inflammatory properties may play a role at the level of endothelium-blood-platelet interaction, thus reducing the procoagulant activity of the inflammatory cytokines. Levels of IL-10 increase significantly in the vein wall during the thrombotic event, suggesting the existence of a compensatory mechanism to counteract pro-inflammatory cytokines [121]. However, at the moment, the exact mechanism by which IL-10 may exert its anti-thrombotic activity is mostly unknown.

## 6. Thromboembolic Mechanisms of Jakinibs

**Figure 3:**
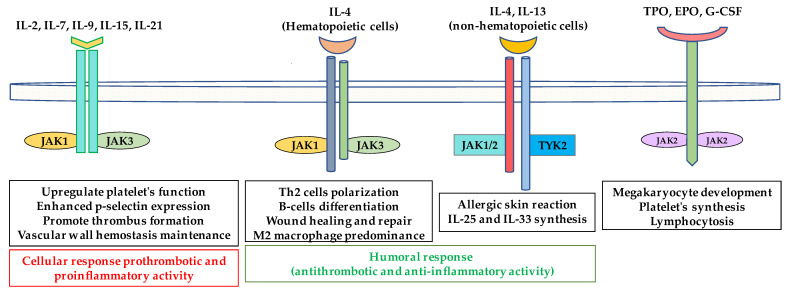
Pro and anti-thrombotic activities of cytokines. Cytokines which signal through the common gamma chain receptor family, and hematopoietins (TPO, EPO and G-CSF) transmit proinflammatory and prothrombotic signals resulting in higher expression of adhesion molecules, vascular wall inflammation and platelet synthesis, which promote thrombus formation. IL-4 and IL-13 are involved in the humoral response, antibody synthesis and lymphocyte polarization toward the Th2 response, thus playing anti-inflammatory roles. Multiple cytokines signal via the same JAK, thus blocking any single JAK may simultaneously inhibit transmission of multiple pro- and anti-thrombotic signals. TPO, thrombopoietin; EPO, erythropoietin; G-CSF, granulocyte-colony stimulating factor.

In the context of thromboembolic risk, targeting, JAK3 and thus parallel inhibition of IL-9 and IL-15 signaling may exert an anti-thrombotic potential as IL-9 and IL-15, through their pro-inflammatory potential, contribute to thrombus formation. In contrast, IL-2, which shares some homology with IL-15, provides contrasting contributions to T-cell-mediated immune responses, and plays a significant role in peripheral tolerance through the elimination of self-reactive T-cells [133]. As all these cytokines utilize the same JAK pairing (JAK1/JAK3, Figure 1) switching off this pathway may cause imbalance between pro-inflammatory and anti-inflammatory activity and thus pro and anti-thrombotic peripheral effects. Theoretically, reducing the immune response at the vascular wall may halt the progression of thrombus formation, however, this reduction will be limited when a Jakinib targeting the anti-inflammatory response more strongly than the pro-inflammatory activity is administered. This effect may be even more pronounced when considering the anti-inflammatory activity of IL-4 and IL-13, which represent the group of typical Th2-dependent (thus anti-inflammatory) cytokines. IL-4 and IL-13 transmit their signals using JAK1/JAK3 and JAK1/JAK2/TYK2 respectively (Figure 1 and Figure 3). IL-4 and IL-13 suppress the release of inflammatory mediators and reduce the production of IL-1β, TNF-α and other pro-inflammatory cytokines [134]. It is currently known that IL-4 inhibits induction of TF thus preventing thrombus formation [135]. Moreover, cerebrovascular events have been reported in the course of treatment of atopic dermatitis with IL-4 and IL-13 antagonists, suggesting an essential role for both cytokines in coagulation system homeostasis [136]. Of note, however, these two cytokines share the same common receptor (IL-4Rα/γ chain and IL-4Rα/IL-13Rα for IL-4 and IL-13 signaling respectively) [137] to signal through different JAK kinase pairings (Figure 3).

Even more contrasting behavior is seen in the IL-10 cytokine family. The family consists of IL-10, a typical anti-inflammatory cytokine and cytokines such as IL-19, IL-20, IL-24 and IL-26 which are referred as the IL-20 subfamily [138] (Figure 2). With the exception of IL-22 and IL-19, IL-20 cytokines exert strong proinflammatory potential (Table 1). The role of IL-22 depends on context, it may exert pro-inflammatory or tissue-protective functions, and this is regulated by the co-expressed cytokine IL-17A. Both IL-10 and IL-22 signal via JAK1/TYK2 whilst the other cytokines in this family use JAK1/JAK2. It is, therefore, plausible that blocking TYK2 and thus IL-10, IL-22, IL-4 and IL-13 signaling may be a potential mechanism via which thromboembolic processes are promoted. This issue, however, should be addressed in future studies (Table 2).

Based on animal and human studies, IFN-γ is a typical pro-inflammatory cytokine with a strong prothrombotic potential [17,72,104]. JAK1 or TYK2 inhibitors may therefore inhibit the pro-inflammatory effect of IFN-γ [149]. However, it is not clear whether blocking the inflammatory response with Jakinibs translates directly to the amelioration of the harmful effects of IFN-γ on thrombus development. Of note is also the fact that both IFNs and the IL-12 family signal through similar JAKs. JAK2 and TYK2 pair for IL-12/IL-23 signal transmission whilst JAK1 pairs with TYK2 or JAK2 to transmit IFN signals [150]. Again, inhibition of TYK2 without any significant reduction of JAK1 and JAK2 activity may facilitate thrombus priming by IL-12/23 and IFN via JAK2 and JAK1, respectively (Figure 1 and Figure 2, Table 1 and Table 2).

Recently, a study in patients with systemic lupus erythematosus showed that baricitinib (a JAK1 and JAK2 inhibitor) reduced levels of IL-6 significantly and also IL12p40 at week 12. Baricitinib treatment did not affect IFN concentration, although the authors reported a reduction in IFN-dependent gene expression [151]. This finding supports the hypothesis that the final result of JAK blockade depends on JAK molecule pairing and is more complicated than targeting one over the other JAK. In this study, baricitinib treatment resulted in down regulation of two inflammatory pathways (IL-6 and IL12 dependent) but left IFN levels unaffected. This may potentially translate to higher thrombotic risk in baricitinib-treated patients as IFN transmits the strongest prothrombotic signals.

As JAK inhibition targets many cytokine pathways simultaneously, we hypothesize that targeting a given JAK results in an imbalance between the various cytokine-dependent pathways. In predisposed patients, this may facilitate the transmission of prothrombotic signals via pathways that are not or are only weakly blocked. That may be especially true as far as currently available Jakinibs are concerned. At the moment, tofacitinib, a pan-Jakinib and baricitinib, which demonstrated selectivity for JAK1 and JAK2 are approved for clinical use. Therefore, when these compounds’ thromboembolic risk is discussed, their specificity should be considered when interpreting these results. Recently, a third Jakinib, upadacitinib, an oral selective JAK1 inhibitor, was approved for the treatment of rheumatoid arthritis by the European Medicines Agency and the US Food and Drug Administration. Results from clinical trials showed that upadacitinib is a potent blocker of IL-4 and IL-13 signaling thus potentially may expert pro-thrombotic effect [152]. However, data on upadacitinib thromboembolic risk are still being collected; therefore, it remains unclear how JAK1 selectivity may modulate this risk [153].

## 7. Clinical Data on Thromboembolic Complications

Since the introduction of tofacitinib, the first Jakinib, to standard clinical practice, Jakinib’s role has continued to grow. However, recently, new data on Jakinibs’ safety regarding thromboembolic complications became available, and a red flag on administration in a specific population of patients has been raised. Venous thromboembolic episodes, including PE and DVT, have been observed in both baricitinib (4 mg daily) [154] and tofacitinib (at both 5 mg and especially 10 mg twice daily) treated patients, particularly in those with elevated risks factors for thromboembolic events and advanced age [155]. Additional data from an analysis of the WHO VigiBase was recently published [156]. The authors stated that classical risk factors (i.e., age, previous thromboembolic episodes and contraceptive use) were directly linked with the development of thromboembolic complications. In line with this finding, Vallejo-Yagüe et al. [156] also supported the previous European Medicines Agency’s recommendations, which suggested that high doses of tofacitinib should be administered with caution (or simply should be avoided) in patients at higher thromboembolic risk (i.e., >65 years of age, concomitant cardiovascular disease, or treated with hormone therapy).

In contrast, the recent study by Poderós et al. [157] suggested that tofacitinib at low to medium dose (10–20 mg daily) may exert a protective role against thromboembolic complications. This has not been observed for baricitinib. These newer findings might be considered controversial as the authors performed only controlled observational studies and clinical trial analysis. Moreover, the findings refer to an idealized trial setting (selected patient populations, inclusion, and exclusion criteria). Whether or not these findings translate to the general population in real-world clinical practice needs to be determined.

The Jakinib’s application is obviously not restricted to rheumatological diseases. Jakinibs have proven to be safe and efficacious in several hematological disorders. At the moment, ruxolitinib and fedratinib; oral Jakinibs with JAK2 selectivity have been approved by the FDA for the treatment of intermediate and high-risk myelofibrosis [158]. The other two Jakinibs, namely, momelotinib and pacritinib are being intensively tested in several clinical trials [159]. As these Jakinibs block selectively JAK2, which results in inhibition of signaling from all three main haemopoietic cytokines (erythropoietin, thrombopoietin and GCSF) a reversible, dose-dependent myelosuppression is the main safety concern. Data from clinical studies, however, did not show higher thromboembolic risk. The same is true for the newer Jakinibs (ilginatinib and itacitinib), which are at the early stages of clinical development, are concerned. As the clinical data for these Jakinibs are limited, at the moment it is still unclear if JAK2 selectivity translates directly to the lower thromboembolic risk.

## 8. Conclusions

All known known cytokines and chemokines create a unique network of self-interactions, activation and regulatory loops. Any disturbance would be predicted to result in the development of autoimmunity, malignancy, allergy, immunodeficiency or immunothrombosis. The JAK/STAT pathway transmits inflammatory signals and is deeply involved in the proper function of many systems of the body as well as the pathogenesis of several autoimmune disorders. Theoretically, inhibition of JAK should translate into reduction of both autoimmune diseases’ activity and reduction of prothrombotic risk. At the moment, it is, therefore, unclear why patients treated with Jakinibs are at higher risk of developing thromboembolic side effects. The clinical situations where Jakinibs should be administered with caution included patients who have experienced previous thromboembolic episodes and those who are taking sex hormone therapy or who are at an advanced age. In these latter instances, we cannot exclude that age-dependent mutation of JAK plays a role. As the frequency of JAK mutation rises with age, it is plausible that prothrombotic mutation may play a role. At the moment, data on the real risk linked with Jakinib administration continues to be gathered. New light will perhaps be shed on this issue when more specific Jakinibs are available in routine clinical practice. We cannot rule out that, contrary to common belief, thromboembolic complications are not a class drug effect, but might be related to JAK inhibition specificity, where an anti-thrombotic pathway is blocked, whilst other pathways that can transmit prothrombotic signals are not affected. This issue should be addressed in the future so as to assess which drug should be administered to a given patient.

## Figures and Tables

**Table 1:** Role of selected cytokines in thrombus formation.

Cytokines	Activities	References
Interleukin-6 family		
IL-6	Prothrombotic	[122]
	Reduction of Vessel Dilation	[123]
	Induces the expression of TF	[124]
	Increases the level of fibrinogen	
	Higher expression of factor VIII	
	High expression of von Willebrand factor	
	Endothelial activation	[92]
	Endothelial cell damage	
	Increased platelet aggregation	
	Increased sensitivity to thrombin	
	Amplify inflammatory response (cytokine storm)	[125]
	Regulate acute phase reactant synthesis	[126]
IL-11	Thrombopoiesis	[127]
Interleukin-10 family		
IL-10	counteract pro-inflammatory cytokines	[121]
IL-22	Dual (pro and anti-inflammatory activity)	[128]
	Potential anti-thrombotic activity?	
Inteleukin-20 subfamily		
IL-19	Expressed in response to vascular injury	[129]
	Exerts a pronounced anti-inflammatory effect upon vascular cells	
IL-20	Strong proinflammatory activity (role in thrombus formation unknown)	[130]
IL-24	Strong proinflammatory potential	[131]
IL-26	Induces production of proinflammatory cytokines by innate immune cells	[132]

**Table 2:** Cytokines signaling through JAK molecules and their effect on thrombus formation.

JAK Type	Cytokine Signaling	Thrombotic Effect	Active Jakinibs
JAK1, JAK2, TYK2		IL-6 family	TOFA (pan-Jakinib) BARI (JAK1/JAK2) UPA (JAK1) RUX, MMD, FEDR, PAC (JAK2)
	IL-6	Proinflammatory and prothrombotic [122]	
	IL-11	Proinflammatory and prothrombotic [127]	
	IL-27	Dual role in inflammation and prothrombotic (?) [139]	
	IL-31	Anti-inflammatory and anti-thrombotic? [140]	
JAK1, TYK2		IL-10 family	TOFA (pan-Jakinib) BARI (JAK1/JAK2) UPA (JAK1)
	IL-10	Anti-inflammatory and anti-thrombotic [119]	
	IL-19	Anti-thrombotic [129]	
	IL-20	Proinflammatory and prothrombotic [130]	
	IL-22	Dual (pro and anti-thrombotic) and proinflammatory [128]	
	IL-24	Prothrombotic and proinflammatory [131]	
	IL-26	Prothrombotic [132]	
JAK1, TYK2 JAK1, JAK2 JAK1, TYK2 JAK1, TYK2		Interferons	TOFA (pan-Jakinib) BARI (JAK1/JAK2) UPA (JAK1)
	IFN-λ	Anti-inflammatory and anti-thrombotic [98]	
	IFN-γ	Proinflammatory and prothrombotic [72]	
	IFN-α	Proinflammatory and prothrombotic [141]	
	IFN-β	Anti-inflammatory and anti-thrombotic [99]	
JAK2, TYK2		IL-12/23	TOFA (pan-Jakinib) BARI (JAK/JAK2) RUX, MMD, FEDR, PAC (JAK2)
	IL-12	Proinflammatory and prothrombotic [116,117]	
	IL-23	Proinflammatory and prothrombotic [116,118]	
JAK2 homodimer		Hematopoietins	TOFA (pan-Jakinib) BAR (JAK1/JAK2) RUX, MMD, FEDR, PAC (JAK2)
	GCSF	Proinflammatory and prothrombotic [142]	
	EPO	Proinflammatory and prothrombotic [143]	
	TPO	Proinflammatory and prothrombotic [144]	
	GMCSF	Proinflammatory and anti-thrombotic (?) [145]	
JAK1, JAK3		γ chain cytokine family	
	IL-2	Possible dual role in inflammation, prothrombotic [146]	TOFA (pan-Jakinib) BAR (JAK1/JAK2) UPA (JAK1)
	IL-4 (IL-4Rα/IL13Rα receptor)	Anti-inflammatory and anti-thrombotic [136]	
	IL-7	Proinflammatory and prothrombotic [147]	
	IL-9	Dual (pro and anti-thrombotic) [148]	
	IL-15	Proinflammatory prothrombotic (?) [146]	
	IL-21		
JAK1, JAK2 TYK2	IL-4 (IL-4Rα/IL-2Rγ receptor)	Anti-thrombotic [136]	TOFA (pan-Jakinib) BARI (JAK1/JAK2) UPA (JAK1) RUX, MMD, FEDR PAC (JAK2)
JAK1, JAK2 TYK2	IL-13	Anti-thrombotic [136]	

TOFA-tofacitinib; BARI-baricitinib; UPA-upacitinib; RUX-ruxolitinib; FEDR-fedratinib; PAC-pacritinib; MMD-momelotinib.

## Abbreviations

RA	Rheumatoid arthritis
DMARDs	Disease-modifying antirheumatic drugs
JAK	Janus kinases
STAT	Signal transducers and activators of transcription
PE	Pulmonary embolism
DVT	Deep vein thrombosis
IFN	Interferon
IL	Interleukin
JH	JAK homology
SOCS	Suppressor of cytokine signaling
GM-CSF	Granulocyte-macrophage colony-stimulating factor
MACE	Major adverse cardiovascular events
VTE	Venous thromboembolic event
